# Niosomal Bupropion: Exploring Therapeutic Frontiers through Behavioral Profiling

**DOI:** 10.3390/ph17030366

**Published:** 2024-03-12

**Authors:** Karthick Harini, Suliman Yousef Alomar, Mohammed Vajagathali, Salim Manoharadas, Anbazhagan Thirumalai, Koyeli Girigoswami, Agnishwar Girigoswami

**Affiliations:** 1 Medical Bionanotechnology, Faculty of Allied Health Sciences (FAHS), Chettinad Hospital & Research Institute (CHRI), Chettinad Academy of Research and Education (CARE), Kelambakkam, Chennai 603103, TN, Indiavajasmash@gmail.com (M.V.); koyelig@gmail.com (K.G.); 2 Doping Research Chair, Zoology Department, College of Science, King Saud University, Riyadh 11451, Saudi Arabia; 3 Department of Botany and Microbiology, College of Science, King Saud University, Riyadh 11451, Saudi Arabia

**Keywords:** depression, niosomes, antidepressant, drug delivery, behavior

## Abstract

Bupropion (Bup) belongs to the norepinephrine–dopamine reuptake inhibitor (NDRI) class and it is the only FDA-approved drug of its class for the treatment of major depressive disorder (MDD), sold under the name of Wellbutrin. Although bupropion is effective in suppressing the symptoms, its regular use and overdose might lead to seizures and liver failure. Thus, we aimed to nanoformulate bupropion onto a niosomal vesicle to improve its efficacy and achieve the same therapeutic effect at lower scheduled doses. A thin film hydration method was adopted to synthesize and optimize Bup entrapped niosomes using three different surfactants of the sorbitan ester series (Span 20, 40, and 60) in combination with cholesterol. The optimization data determined that the niosome formulated with a cholesterol-to-surfactant ratio of 1:1.5 is the most stable system, with the Bup entrapped niosomes containing Span 20 (Bup@N_20_C) exhibiting minimal in vitro and in vivo toxicity, and demonstrating the sustained release of Bup in artificial cerebrospinal fluid (ACSF). The Bup@N_20_C formulation showed increased exploration activity and reduced irregular movements in reserpine-induced depression in the adult zebrafish model, suggesting the potential for mood improvement through the suppression of depression-like behavior which was established by statistical analysis and trajectory data. The Bup@N_20_C-treated group even surpasses the treatment effect of the positive control group and is comparable to the control group. Hence, it can be inferred that niosomal formulations of Bup represent a promising delivery system capable of achieving the brain delivery of the cargo by bypassing the blood–brain barrier facilitated by their small architectural structure.

## 1. Introduction

According to the World Health Organization (WHO), around 3.8% of the population is affected by major depressive disorder (MDD) or simply depression globally, of which women suffer from MDD more compared to men [1]. Depression is considered a chronic and recurrent illness, which mainly occurs due to neurobiological events, that is, the sudden drop in the level of neurotransmitters, while stressful events, chronic medical conditions, and side effects from other medications such as beta-blockers are also contributing factors [2,3]. When discussing neurobiology, all three systems under it (neurochemistry, neuroanatomy, and neuroimmunology) are equally important. The brain is a complex system and it is not fully understood, and treating any condition related to it is burdensome [4,5,6]. Neurotransmitters (NTs) are important signaling molecules that mainly account for communication. The relationship and imbalances between monoamine NTs such as dopamine (DA), 5-hydroxytryptamine, or serotonin (5-HT), and norepinephrine (NA) are the notable neurochemistry of depression [7,8]. Joseph Jacob Schildkraut explained this under a theory called ‘The Monoamine Hypothesis’ [9]. Understanding the role of NTs in MDD is crucial since every NTs plays a different role in the pathophysiology of the disease condition [10].

Certain anatomical structures of the brain, especially the limbic system comprising the cingulate gyrus, amygdala, and hippocampus, play a crucial role in MDD. Several pieces of evidence reported the connection between these structures and the behavioral activities of an individual, but the exact mechanism behind them is not well established due to the lack of studies with advanced neuroimaging systems [11,12]. The immunological mechanisms are other dominant factors in the pathogenesis of MDD. Cytokines, the key immune signaling molecules at higher concentrations in the body, greatly contribute to the pathogenesis of MDD. These increased levels of cytokines have the capability to deplete the peripheral tryptophan, the major precursor of 5-HT, causing significant neurochemical changes, in turn leading to lower levels of 5-HT secretion [13]. The prolonged exposure to high concentrations of the inflammatory cytokines may affect the function of glucocorticoid receptors [14]. Osimo et al. reported a meta-analysis study on cytokine alterations and their variability in patients with depression [15]. The analysis results declared that depression in acute conditions is a pro-inflammatory state. Besides these neurobiological aspects, genetics has also been reported to have great implications. More than 200 genes have been identified as responsible for the occurrence of MDD. Examining multiple hereditary analyses underscores a consistent finding: a higher susceptibility to MDD in women compared to men. This aligns with the earlier assertion. However, the complexity of comparing hereditary information arises from variations in the selected study population, employed instruments, conceptual frameworks, and methodologies [16]. 

Cognitive behavioral therapy (CBT) is mostly the preferred therapy when the individual is diagnosed with acute depression. This therapy involves discussing the negative thoughts and destroying them by making the patient understand their surroundings. The main issue here is the cooperation and understanding of the patient. A patient needs to develop trust in the therapist before opening up their personal issues, which sometimes fails [17,18,19,20]. Psychoanalytical therapy is a technique where the therapist explores the unconscious state of mind with inner conflicts and the way it affects the conscious process. However, this therapy is regarded as a long-term process since the therapeutic result of this therapy is time-consuming and not very effective [21,22]. Various groups of drugs that could lift the mood and improve motivation are classified as antidepressants. Antidepressants were developed based on the monoamine theory and they work by inhibiting the reuptake of neurotransmitters. The first class of antidepressants is monoamine oxidase inhibitors (MAOIs), which work by increasing the concentration of 5-HT, DA, and NA by inhibiting the breakdown of these NTs caused by monoamine oxidase type A (MAO-A) and type B (MAO-B) [23,24]. The tuberculostatic agent iproniazid was later found to stimulate the central nervous system (CNS), elevate mood, and improve the concentration of 5-HT. Therefore, iproniazid was planned to be used for the treatment of MDD and became the first MAOI. Even though the recovery rate was satisfactory, it displayed non-selective binding, causing liver damage and disturbing the blood pressure condition by tyramine concentration [25,26]. Hence, a more selective novel class of drug called RIMA, which could reversibly inhibit the MAO-A, was developed [26]. The MAOIs’ drug interaction with tyramine concentration was an unsolved issue, so patients under the medication are advised to avoid tyramine-containing foods [27]. Tricyclic antidepressants (TCAs) were then developed, which also target the degradation of monoamine NTs, similarly to MAOIs. They function by boosting the activity of the transporters of 5-HT and NA by desensitizing the presynaptic receptors [28]. This desensitization is performed to accumulate the 5-HT and NA NTs in the presynaptic cleft, which is achieved by inhibiting the reuptake transporters. TCAs encounter several adverse effects since they also function as competitive antagonists of histamine, muscarinic, adrenergic, and postsynaptic receptors [29]. 

Other medications, such as selective serotonin reuptake inhibitors (SSRIs) and serotonin and norepinephrine reuptake inhibitors (SNRIs), are more effective than TCAs, and, so, the use of TCAs was not much considered until and unless required [30]. The SSRIs and SNRIs, from the time of development till today, are considered the first line of treatment for MDD. However, the adverse effects of SSRIs involve gastrointestinal disorders, gynecomastia, sexual dysfunctions, and agitation, and SNRIs involve the dysregulation of the metabolic processes of hyperglycemia [31]. The onset of action of drugs belonging to either class is delayed. Recently, the FDA approved gepirone hydrochloride, which acts as an agonist of the 5-HT 1a receptor for the treatment of MDD [32]. But for the cases that report on the issues with NA and DA NTs, drugs belonging to the norepinephrine and dopamine reuptake inhibitor (NDRI) class are prescribed [33]. Unfortunately, there is only one bupropion drug in the class that has been accepted by the FDA for clinical use [34]. NDRIs work by blocking the transport of DA and NA back into the brain cells. NDRIs are considered as atypical antidepressants since the mechanism of action is completely different from the other class of antidepressants. Although drugs that belong to the SSRI class are preferred as the first-line medication, bupropion is given as an adjuvant when patients fail to respond to SSRIs and is more effective than them [35]. However, there is always a search for newer and more effective solutions. NDRI drugs are considered effective but patients who particularly require NDRI drugs are left with no choice other than bupropion. At the same time, bupropion is reported to cause seizures, cardiac failures, and other allergic reactions at high doses, as it also develops addiction [36]. Developing a novel drug is a great burden as the time and money it requires are huge. Another great challenge of any antidepressant is to pass through a microvasculature structure of the CNS called the blood–brain barrier (BBB), which is a most selective semi-permeable membrane located in the third and fourth ventricles of all the brain regions, and at times some antidepressants fail to do so [37]. The interaction study by O’Brien et al. found that the blockage of drug entry through the BBB is mostly caused by the P-glycoprotein in the BBB [38]. Alavijeh et al. reviewed the crucial role of the BBB in CNS-related drug delivery [39]. 

Nanomedicine is a wise choice, as drugs made in the nanoscale eliminate adverse effects to a greater extent compared to pure drugs [40]. The advantage of nanoformulated drugs in CNS delivery is that they can easily escape the barrier system, as well as improve solubility, enable targeted delivery, provide sustained release, and open up the possibility of diagnosis while ensuring the safety and efficacy of the drug with minimal side effects [39]. Jani et al. prepared polymeric nanocarriers to deliver agomelatine via the intranasal route of delivery [41]. Nanoprecipitation was adopted for the preparation of the formulation. Upon nanoformulation, the permeability observed in the ex vivo studies was higher than that of the drug alone. To assess the route of administration, the goat nasal mucosa was used and no evident nasal toxicity was observed. The antidepressant activity of the formulation was confirmed by studying the animal model. Of all nanoparticles, nanovesicles, especially niosomes, are of great interest as a drug vehicle [42]. Niosomes can gain easy access to the brain by bypassing the BBB [43]. Since the role of nanomedicine for CNS delivery is well documented, nanoformulating antidepressants is a major research field [44]. Gupta and colleagues devised a transdermal delivery method for the poorly soluble medication, sertraline, employing various transfersomal formulations comprising non-ionic surfactants [45]. Their findings demonstrated the successful entrapment of sertraline in all formulations with consistent drug content. The optimized formulation was determined to be a transfersomal gel containing 1.6% of the drug and 20% of Span 80, exhibiting the highest drug entrapment at 90.4% and a cumulative drug release of approximately 73.8%. Brain research, especially depression-related studies, often involves the zebrafish model, as the neurotransmitter system of zebrafish is very similar to humans and contains all the NTs that account for MDD [46]. The zebrafish also shares genetic similarities with humans, including stress response and neuroanatomy [47]. Zhang et al. compared the reversal of induced-depression symptoms in sertraline- and herbal-medicine-treated zebrafish models [48]. Behavioral studies found that the herbal medicine cured the depression condition better than the commercially available sertraline. Indeed, while niosomes offer advantages such as non-toxicity, enhanced chemical stability, and surface modification flexibility, challenges persist [49,50,51]. These include optimizing drug encapsulation, reducing unencapsulated drugs, ensuring stability in the cerebrospinal fluid, and standardizing key components. 

This research focuses on utilizing niosomes to nanoformulate bupropion, intending to create a more advantageous treatment approach for individuals in need of NDRI drugs, and simultaneously seeks to address the significant adverse effects typically associated with bupropion usage through this innovative nanoformulation strategy. Usually, the intranasal route is highly preferred for the delivery of therapeutics to the brain when studied in rodent models. This research is conducted by taking zebrafish (ZB) as the neuro model and we preferred the oral route for the delivery of the formulation. In addition, the major issue regarding the nanoformulation is its stability. Our work has addressed the issue by assessing the stability of the prepared formulation. The nanoformulation of bupropion has not yet been reported. To our knowledge, this is the first study that attempted to nanoformulate bupropion delivered orally to study its therapeutic efficacy with behavioral studies in vivo using the zebrafish model. The study includes optimization of the cholesterol and surfactant concentration, and the optimized vesicles are used for further experiments. The optimized vesicles are denoted N_20_C, N_40_C, and N_60_C for Span 20, 40, and 60, respectively, while the bupropion-loaded Span 20, 40, and 60 vesicles will be denoted as Bup@N_20_C, Bup@N_40_C, and Bup@N_60_C, respectively. The other formulation codes of the vesicles are described in the Supplementary Materials.

## 2. Results and Discussion

### 2.1. Optimization of Cholesterol Concentration

Niosomes containing various concentrations of cholesterol were prepared to study their varying concentration effect on the encapsulation to ensure successful entrapment of bupropion (Bup) and to obtain the optimum concentration suitable for the study. The effect of cholesterol on the absorbance and particle size was studied using a UV–visible spectrophotometric and dynamic light scattering technique, respectively.

#### 2.1.1. Effect of Cholesterol on the Absorbance

**Figure 1:**
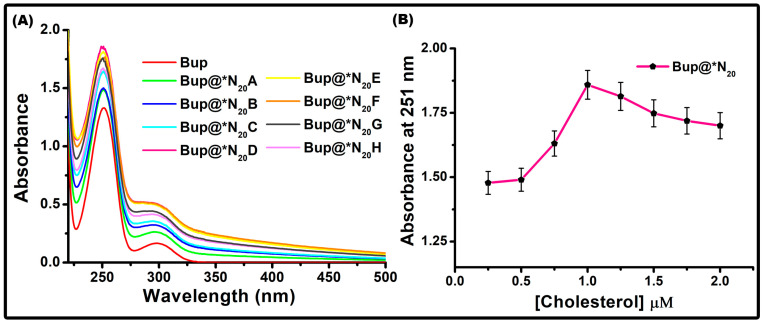
Optimization of the cholesterol concentration using bupropion as an external probe in a Span 20 vesicle. Effect of cholesterol concentration on absorbance. (**A**) Absorption spectra of bupropion encapsulated in Span 20 niosomal vesicles. (**B**) OD at 251 nm plotted against variable cholesterol concentrations.

Cholesterol, the most recognized sterol molecule, is amphiphilic due to the presence of a hydrophobic hydrocarbon domain and hydrophilic 3-hydroxy group. It is usually added during the synthesis of niosomes, as it provides stability to the structure by preventing aggregation, decreasing the fluidity of the membrane structures, and minimizing the leakage of the payload by keeping the structure intact [52]. Cholesterol is positioned in the bilayer structure of niosomes by forming hydrogen bonds with the hydrophilic head of the surfactant. This hydrogen bonding with the surfactant is facilitated by their hydroxyl group [43]. During the synthesis, under a vacuum rotary evaporator, turbidity was noted, which was considered as the preliminary confirmation of vesicle formation. We used Bup as an external probe to study the effect of cholesterol concentration on niosomes made of Span 20. The bare Bup showed a maximum absorption at 251 nm and a second peak appeared at 298 nm (Figure 1A). After drug loading, the absorbance of all the vesicles at varying concentrations of cholesterol was recorded and is represented in Figure 1A. Figure 1B shows the graphical representation of the OD obtained at 251 nm against variable concentrations of cholesterol. The recorded optical density at 298 nm was marginally lower and, consequently, omitted from plotting, but a similar observation was evident at 251 nm. The Bup-loaded vesicles showed a similar absorption behavior to bare Bup but, during the analysis, we observed something interesting. In the preliminary stages, the augmentation in absorbance correlated with an ascending cholesterol concentration, thereby manifesting a discernible alteration in the microenvironment surrounding Bup, suggestive of its encapsulation or entrapment within niosomes. However, at a point after reaching the maximum absorption, the absorbance started to decrease at a very slow rate with further increasing concentrations of the cholesterol components in the niosomal structures. One could hypothesize that the elevated cholesterol concentration may facilitate hydrophilic interactions with the drug, thereby impeding its encapsulation. The highest OD of encapsulated Bup was noted in the vesicle with a cholesterol concentration of 1 μM, leading to the establishment of 1 μM as the optimal cholesterol concentration for the final niosome formulations.

#### 2.1.2. Effect of Cholesterol on the Particle Size

The size of niosomal vesicles typically fluctuates with variations in the concentration or molar ratio of the cholesterol and surfactant. This variation arises from their involvement in packing density, as they competitively vie for space within the bilayered structure. Nevertheless, the reduction in the niosome diameter accompanying an increase in the cholesterol concentration can be elucidated by considering bilayer hydrophobicity and surface free energy [53]. An excess of cholesterol tends to enhance hydrophobic behavior, subsequently leading to a decrease in surface free energy.

**Figure 2:**
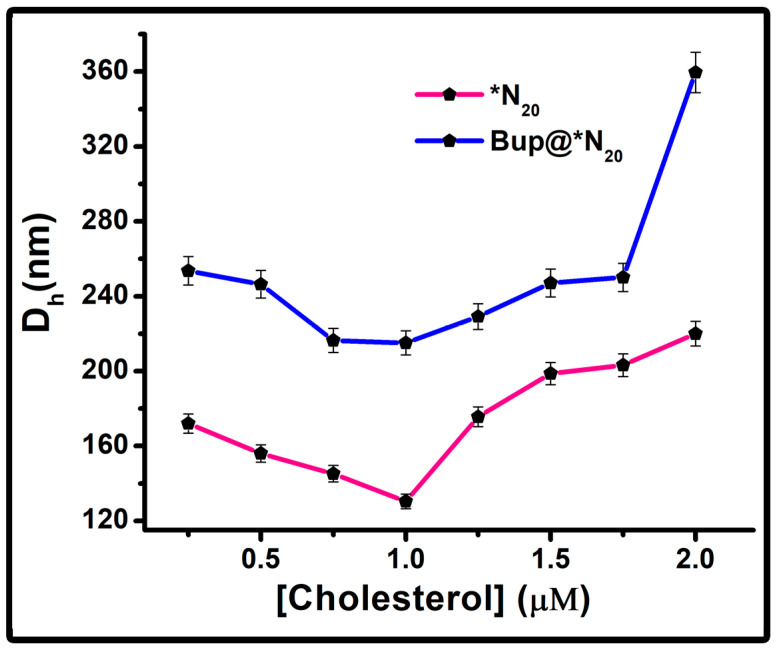
Representation of colloid particle size distribution with scattered plot of N_20_ and Bup@N_20_ with varying cholesterol concentration.

Due to this reason, when the cholesterol concentration was increased, the size of the Span 20 niosomes was dropped, as shown in Figure 2. All the Bup encapsulated vesicles showed an increase in size compared to the empty vesicles, confirming the encapsulation (Table S2 in Supplementary Materials). The vesicle with the cholesterol concentration 1 μM showed the smallest D_h_ of 130.4 nm, which upon encapsulation of bupropion showed D_h_ of 215.1 nm. This result matched the spectrophotometric observation. Hence, the optimum cholesterol concentration is fixed as 1 μM for further experiments. The hydrodynamic diameter exhibits an increase beyond a 1 μM concentration of cholesterol, presumably attributed to the presence of loosely bound cholesterol on the surface of the niosomes. The spectral representation of the D_h_ of the cholesterol-optimized empty vesicle and drug-encapsulated niosomal vesicle is given in the Supplementary Materials (Figure S3 in Supplementary Materials).

### 2.2. Optimization of Surfactant Concentration

**Figure 3:**
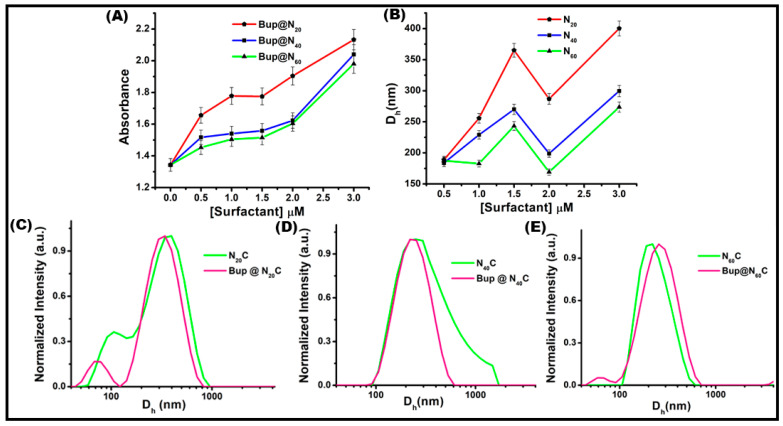
Optimization of the surfactant concentration using Bup as an external probe in Span 20, 40, and 60 vesicles. Effect of surfactant concentration on absorbance. (**A**) Scatter plot representation of OD at 251 nm of Bup@N_20_, Bup@N_40_, and Bup@N_60_. (**B**) Representation of colloid particle size distribution in the scatter plot of varying surfactant concentration of N_20_, N_40_, and N_60_. Spectral representation of the hydrodynamic diameter of (**C**) N_20_C and Bup@N_20_C, (**D**) N_40_C and Bup@N_40_C, and (**E**) N_60_C and Bup@N_60_C.

Nonionic surfactants consist of polar and non-polar components. The determination of whether bilayer vesicles or micelles form is contingent upon the hydrophilic–lipophilic balance (HLB) of the particular surfactant, the chemical structure of the surfactants, and the critical packing parameter. The critical packing parameter is articulated through the ratio of the volume of the hydrophobic group (V) to the area of the hydrophilic head group (A), which is multiplied by the critical length of the hydrophobic group (L) or, mathematically, V/Al [54,55]. The nature and chemical structure of surfactants play a crucial role in promoting the self-assembling niosome formation and entrapment of drugs in it. With the fixed 1 μM concentration of cholesterol using Span 20 niosomal vesicles, an attempt was made to find the optimal surfactant concentration by studying the effect of the surfactant on the absorbance and particle diameter using a UV–visible spectrophotometer and particle-size analyzer, respectively. Figure 3A shows the scatter plot of the OD recorded at 251 nm of all three surfactants at varying concentrations. The OD at zero is the absorbance of bare Bup. With the elevation of surfactant concentration, a rise in absorbance was observed. This occurred because an increase in the surfactant concentration resulted in enhanced niosome formation, leading to a greater entrapment of the drug. A plateau was evident at a surfactant concentration of 1 μM, which persisted until 1.5 μM. This suggests the optimal formation of niosomal vesicles and drug entrapment at 1.5 μM. Upon further increasing the concentration, nearly doubling that of cholesterol, the likelihood of niosome formation decreased, raising the possibility of aggregated or micellar structures. This trend was reflected in the absorbance of Bup. In the micellar structure, the hydrophobic tail faces inward, while the hydrophilic head is exposed. During this, the drug binds to the structure through electrostatic interaction. This is why the absorbance increased as we increased the surfactant concentration. For the other surfactants, a similar observation was recorded. But, unlike Span 20, the absorbance in Span 40 and Span 60 vesicles was less. This can be explained in terms of the chain length and HLB of the surfactants. All three surfactants contain the same head group but different tail groups. The Span 20 surfactant here showed higher absorbance than the other surfactants due to its short chain length and high HLB value of 8.6, while the Span 60 surfactant with a long chain and low HLB value of 4.7 showed low absorbance compared to the others. In contrast to Span 20, the plateau was noted at 0.5 μM concentration in Span 40. The probability of niosome formation at a low concentration of Span 40 is high as it has a lower HLB of 6.7 than Span 20. The optimal surfactant concentration was established at 1.5 μM as, beyond this threshold, a sudden increase in absorbance was observed, primarily attributed to the formation of micelles or aggregates.

Figure 3B shows the scatter plot representation of the D_h_ of the vesicles made with varying surfactant concentrations. In the dynamic light scattering (DLS) study, the observed changes aligned with the spectrophotometric findings. As the surfactant concentration increased, there was a corresponding increase in the niosomal vesicle size. Once the maximum diameter was reached, a sudden decrease was observed. This reduction in diameter corroborated the earlier spectrophotometric results, indicating the aggregation of surfactant molecules into smaller aggregates. Subsequently, as the surfactant concentration further increased, the hydrodynamic diameter increased, which can be potentially attributed to the formation of a larger number of aggregates. The optimal vesicle diameter was observed in the niosomal vesicles with a surfactant concentration of 1.5 μM, leading to the conclusion that the optimized surfactant concentration is 1.5 μM. Figure 3C–E are the spectral representations of the D_h_ of the optimized empty and drug-loaded vesicles prepared with surfactant Span 20, Span 40, and Span 60, respectively. Upon drug encapsulation, there was a notable reduction in the diameter of the vesicles. The binding of Bup to the niosomal vesicles through hydrophilic interactions played a role in this size reduction. Additionally, the rigorous sonication employed for drug entrapment could be another contributing factor to the decrease in the hydrodynamic diameter for Bup@N_20_ and Bup@N_40_. This alteration in the hydrodynamic diameter following drug encapsulation serves as further evidence of Bup entrapment to the niosomes. The major scattering peaks for niosomal vesicles composed of Span 20, Span 40, and Span 60 were noted at 365.1 nm, 256 nm, and 243.2 nm, respectively, indicating their respective D_h_, whereas Bup encapsulated niosomes showed major scattering peaks at 344 nm, 249 nm, and 276.6 nm, respectively, for Bup@N_20_, Bup@N_40_, and Bup@N_60_ nm. The average D_h_, PDI, and zeta potential values of all the formulations were recorded and are presented in Table S2. The PDI value of all the samples was less than 0.7 and confirmed the monodispersity, while the zeta potential measurement showed that the prepared nanoformulation was highly stable as a colloidal suspension. The vesicles with 1:1.5 cholesterol: surfactant concentration were used for further experiment and will be denoted as N_20_C, N_40_C, and N_60_C, while the bupropion-loaded vesicles will be denoted as Bup@N_20_C, Bup@N_40_C, and Bup@N_60_C.

### 2.3. FTIR Analysis

**Figure 4:**
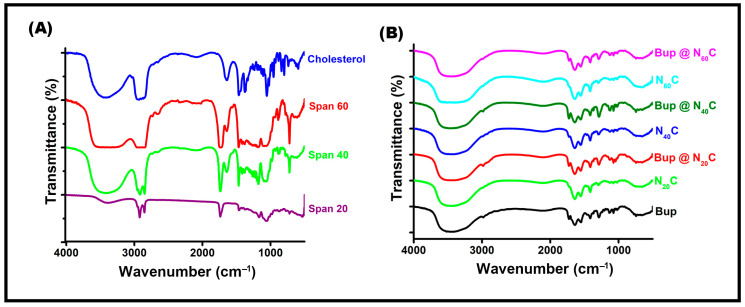
FTIR spectra of (**A**) surfactants and cholesterol, (**B**) empty niosomes and bupropion-loaded niosomes.

For further confirmation of the niosome formation and encapsulation, the chemical bonding between the nanoparticle and bupropion was studied using FTIR spectroscopy. The characteristic peaks were measured in the range of 4000 to 500 cm^−1^ by drying the liquid sample and preparing a pellet. The FTIR spectra of the cholesterol surfactants are shown in Figure 4A, while the bare bupropion, empty vesicles, and bupropion-loaded vesicles prepared with all three surfactants are shown in Figure 4B. The characteristic peak of cholesterol was observed at 3400 cm^−1^, 3000–2850 cm^−1^, 1470–1450 cm^−1^, and 1055 cm^−1^, which corresponds to O-H stretching, C-H stretching, C-H bending, and C-O stretching, respectively [56]. The Span 20 surfactant presented major peaks at 3395 cm^−1^, 2922 cm^−1^, 2852 cm^−1^, and 1737 cm^−1^ that represent -OH stretching, -CH- asymmetric stretching, -CH- symmetric stretching, and C=O stretching, respectively. The Span 40 surfactant showed diagnostic peaks at 3400 cm^−1^, 1737 cm^−1^, and 1470 cm^−1^ due to OH stretching, C=O stretching, and C-H bending, respectively. The Span 60 surfactant spectra showed broad peaks at 3100–3600 cm^−1^, 1737 cm^−1^, 2852 cm^−1^, and 721 cm^−1^ corresponding to OH stretching, C=O stretching, -CH- symmetric stretching, and –CH_2_ rocking, respectively. The characteristic peak of the vesicles featured a broad peak in the range of 3000–3700 cm^−1^, demonstrating strong hydrogen bonding between the surfactants and cholesterol. We observed changes in the spectral structure of the vesicle when loaded with the bupropion. Pure bupropion showed a peak around 2983 cm^−1^ due to the C-H stretch, which was present in all bupropion-encapsulated vesicles with a mild shift and structured and demonstrated successful encapsulation. The spectral regions showed pronounced changes, indicating the interaction between the polar head groups of the surfactants and the drug molecules. Hydrogen bonding and hydrophobic interactions might play an important role in this drug entrapment strategy, and the FTIR spectra are the signature for the formation of the niosomes and successful encapsulation of the bupropion.

### 2.4. Stability Studies

**Figure 5:**
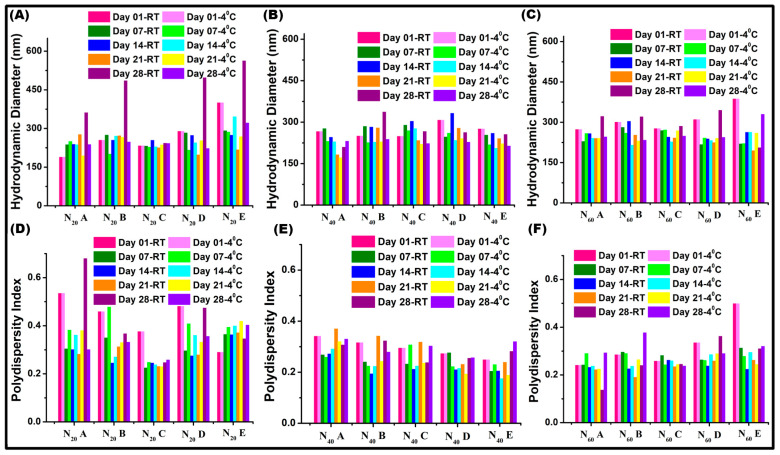
Stability of the vesicle was analyzed by recording the hydrodynamic diameter and PDI in different time intervals (0, 7, 14, 21, 28 days). (**A**,**D**) show the change in D_h_ and PDI of N_20_ vesicles, (**B**,**E**) show the change in D_h_ and PDI of N_40_ vesicles, (**C**,**F**) show the change in D_h_ and PDI of N_60_ vesicles.

The stability of the vesicle was studied for a period of 28 days while the size and polydispersity index (PDI) were recorded on every seventh day of storage. Some variations in both D_h_ and PDI were observed in the samples stored at room temperature (25 °C), while the samples kept in the refrigerator (4 °C) showed comparatively very low variations [57]. Among the three vesicles, the Span 20 surfactant-based vesicles stored in both temperature conditions exhibited no obvious changes in PDI and D_h_. The stability study results of the sample stored at both temperatures showed a minimum effect on the vesicle size and PDI prepared with a cholesterol: surfactant concentration of 1:1.5 (Figure 5), which was consistent with the spectrophotometer and DLS observations.

### 2.5. Surface Morphology, Release Kinetics, and Encapsulation Efficiency

**Figure 6:**
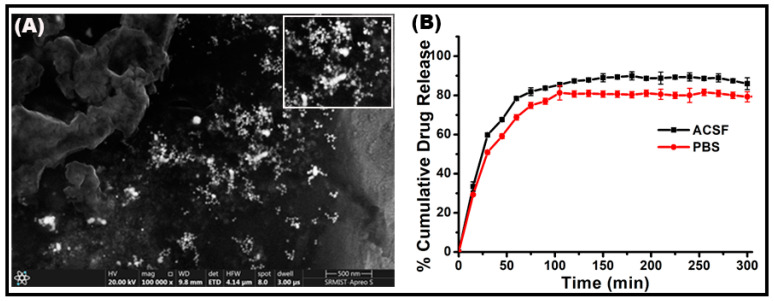
(**A**) Scanning electron microscopic image of Bup@N_20_C (insert: zoomed portion). (**B**) Cumulative percentage drug release vs. time plots of bupropion from N_20_C vesicle in PBS and ACSF medium.

The scanning electron microscopic analysis of Bup@N_20_C showed a spherical morphology with a size of 62 nm (Figure 6A). The release profile of bupropion in the N_20_C vesicle is shown in Figure 6B. During the first few hours, there was a burst release. This may be due to the unbound drug molecules present on the surface of the vesicle. After a point in time, a sustained release of the bupropion was noted. Compared to the release behavior of Bup@N_20_ in PBS, it showed better release in the ACSF dissolution medium. As the ACSF consists of almost all the components of the human CSF, it can be considered as an exact mimic. So, the sustained release behavior of Bup@N_20_ in the ACSF will greatly contribute to enhancing the therapeutic index. The EE % of Bup@N_20_C, Bup@N_40_C, and Bup@N_60_C was found to be 29.1%, 33.1%, and 49%, respectively, and the length of hydrophobic tails plays a major role in the same.

### 2.6. Behavioral Analysis of Adult Zebrafish

**Figure 7:**
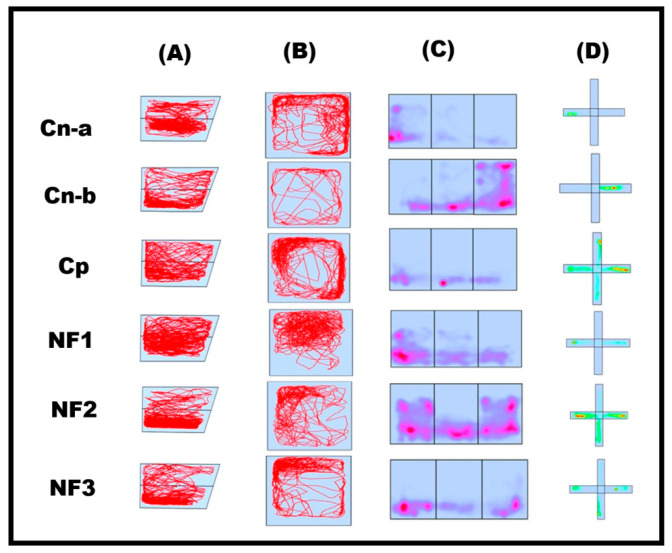
The trajectory data of adult zebrafish. Two-dimensional reconstruction of swimming patterns in (**A**) NTT and (**B**) OFT, (**C**) heat plot of swimming patterns in SPT, and (**D**) Color Maze.

The antidepressant activity of the nanoformulated bupropion was studied by analyzing the behavioral activities of the adult zebrafish (ZB). Figure 7 describes the trajectory data of the fish obtained using ANY-maze software version (7.37). The negative control (healthy fishes (Cn-a)) group of fishes exhibited a wide spatial range and a dense trajectory. The negative control (reserpine-induced depression (Cn-b)) group displayed hypoactivity, reduced exploration, and social avoidance, confirming the successful induction of depression-like behavior using reserpine. The Cp group (positive control) was induced with depression and treated with the commercial antidepressant bupropion. NF1, NF2, and NF3 represent the study groups, i.e., depression induced and treated with Bup@N_20_C, Bup@N_40_C, and Bup@N_60_C, respectively.

**Figure 8:**
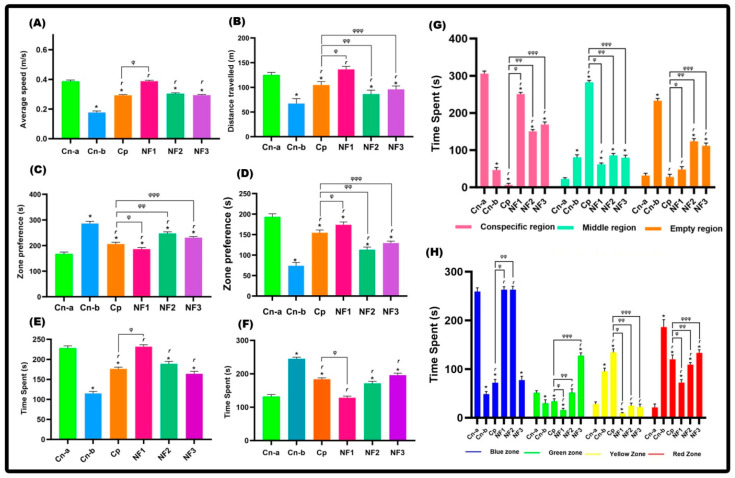
Histograms of the locomotion profile. (**A**) Average speed. (**B**) Total distance traveled. (**C**) Time spent in the upper region. (**D**) Time spent in the lower region. (**E**) Preference for the dark region. (**F**) Preference for the light region. (**G**) Social preference. (**H**) Preference for color. The symbol ‘*’ represents a significant difference between the control and the other groups. The symbol ‘r’ represents a significant difference between the reserpine-treated group and the other groups. The symbols ‘φ’, ‘φφ’, and ‘φφφ’ represent significant differences between positive control and NF1, NF2, and NF3, respectively.

The Cp group of fish showed improved behavioral activity after the treatment. Compared with the Cp group, the study groups showed significant improvement. Among the study groups, the NF1 group displayed an enhanced therapeutic effect, which we predict was because of the vesicle stability and sustained release of the bupropion. Even though the Cp group relieved the state of depression, the NF1 group brought back a behavioral state similar to that of the Cn-a group. At the same time, no notable adverse effects were recorded in the study groups NF1 and NF2. Figure 8 represents the swimming behavior data expressed by mean ± SEM (standard error of the mean). However, one fish from NF3 and one fish from the Cp group died after 9 days of treatment. So, the nanoformulated bupropion (NF1) works better and faster compared to the pure bupropion. The statistical analysis calculated by employing one-way ANOVA also supports the same (Table S3 in Supplementary Materials).

### 2.7. In Vitro Toxicity Assessment

**Figure 9:**
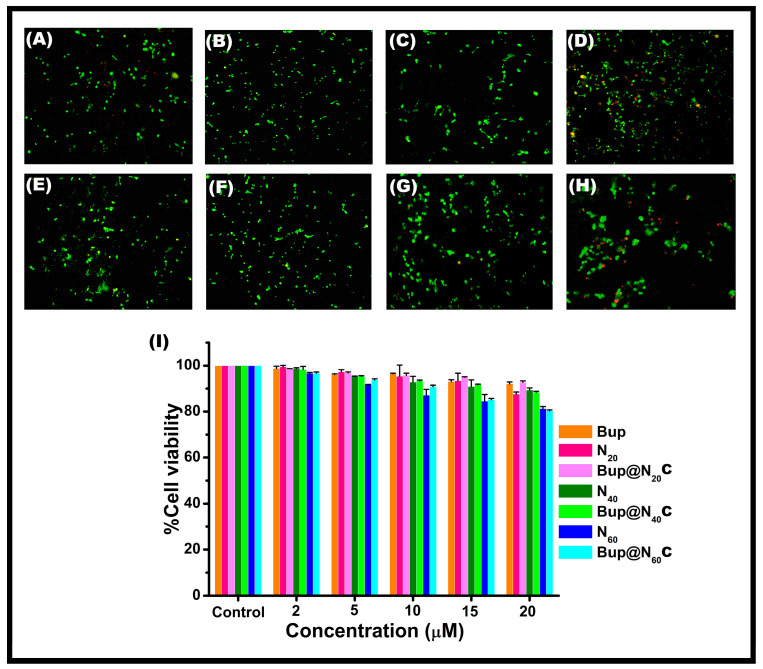
AO/Ebr-stained V79 cells under fluorescence microscope: (**A**) control, (**B**) cells treated with N_20_C, (**C**) cells treated with N_40_C, (**D**) cells treated with N_60_C, (**E**) cells treated with Bup, (**F**) cells treated with Bup@N_20_C, (**G**) cells treated with Bup@N_40_C, (**H**) cells treated with Bup@N_60_C. (**I**) The cell viability assessment using MTT for N_20_C, N_40_C, N_60_C, Bup, Bup@N_20_C, Bup@N_40_C, and Bup@N_60_C after treatment with varying concentrations.

**Figure 10:**
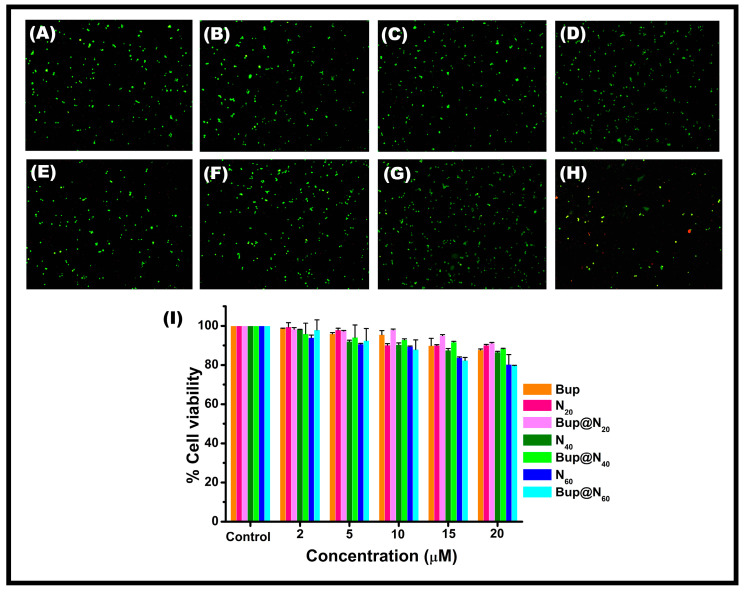
Fluorescence microscopic images of AO/Ebr-stained PC12 cells: (**A**) control, (**B**) cells treated with N_20_C, (**C**) cells treated with N_40_C, (**D**) cells treated with N_60_C, (**E**) cells treated with Bup, (**F**) cells treated with Bup@N_20_C, (**G**) cells treated with Bup@N_40_C, (**H**) cells treated with Bup@N_60_C. (**I**) The cell viability assessment using MTT after treatment with varying concentrations of N_20_C, N_40_C, N_60_C, Bup, Bup@N_20_C, Bup@N_40_C, and Bup@N_60_C.

The V79 (Figure 9) and PC12 (Figure 10) cells treated with varying concentrations of the synthesized vesicles and drug-encapsulated vesicles showed a cell viability of above 85% in all formulations. The results suggest that the cells treated with Bup@N_20_C showed a higher cell viability than the other formulations, while all the empty vesicles showed almost no toxicity. Comparatively, the Bup entrapped Span 60 vesicles were a little toxic and showed a viability of around 80 ± 1% in V79 cells and 79 ± 4% in PC12 cells. The highest concentration of the formulations was treated to perform a live/dead cell assay. The live cells appeared green by taking up the AO, while the dead cells appeared red by taking up the EBr due to poor membrane integrity. The N_20_C vesicles and Bup@N_20_C formulation showed superior cell viability.

### 2.8. Evaluation of Hemocompatibility

**Figure 11:**
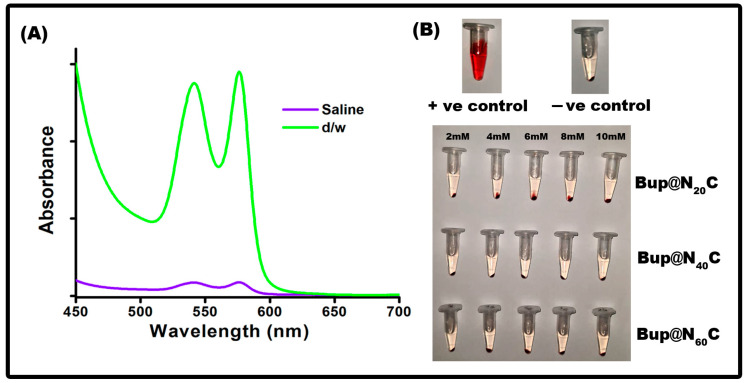
Hemocompatibility at different concentrations (2, 5, 10, 15, and 20 µM). (**A**) Hemocompatibility of the positive and negative control for Bup@N_20_C. (**B**) Photograph of samples incubated with variable concentrations of Bup@N_20_C, Bup@N_40_C, and Bup@N_60_C.

The hemocompatibility of the formulations is investigated since it needs to be administrated into an in vivo system using a hemolysis assay. With varying concentrations, the OD was measured spectrophotometrically and analyzed using the formula. According to the standard test method for the analysis of the hemolytic products of nanoparticles (ASTM E252408), the nanoparticle that exhibits a hemolysis rate of less than 5% can only be administered in an in vivo system [47]. The rate of hemolysis in all the formulations was less than 2%. Hence, it is concluded that the synthesized particle displayed superior compatibility in the assay (Figure 11).

### 2.9. Toxicity Assessment in Zebrafish Embryos

**Figure 12:**
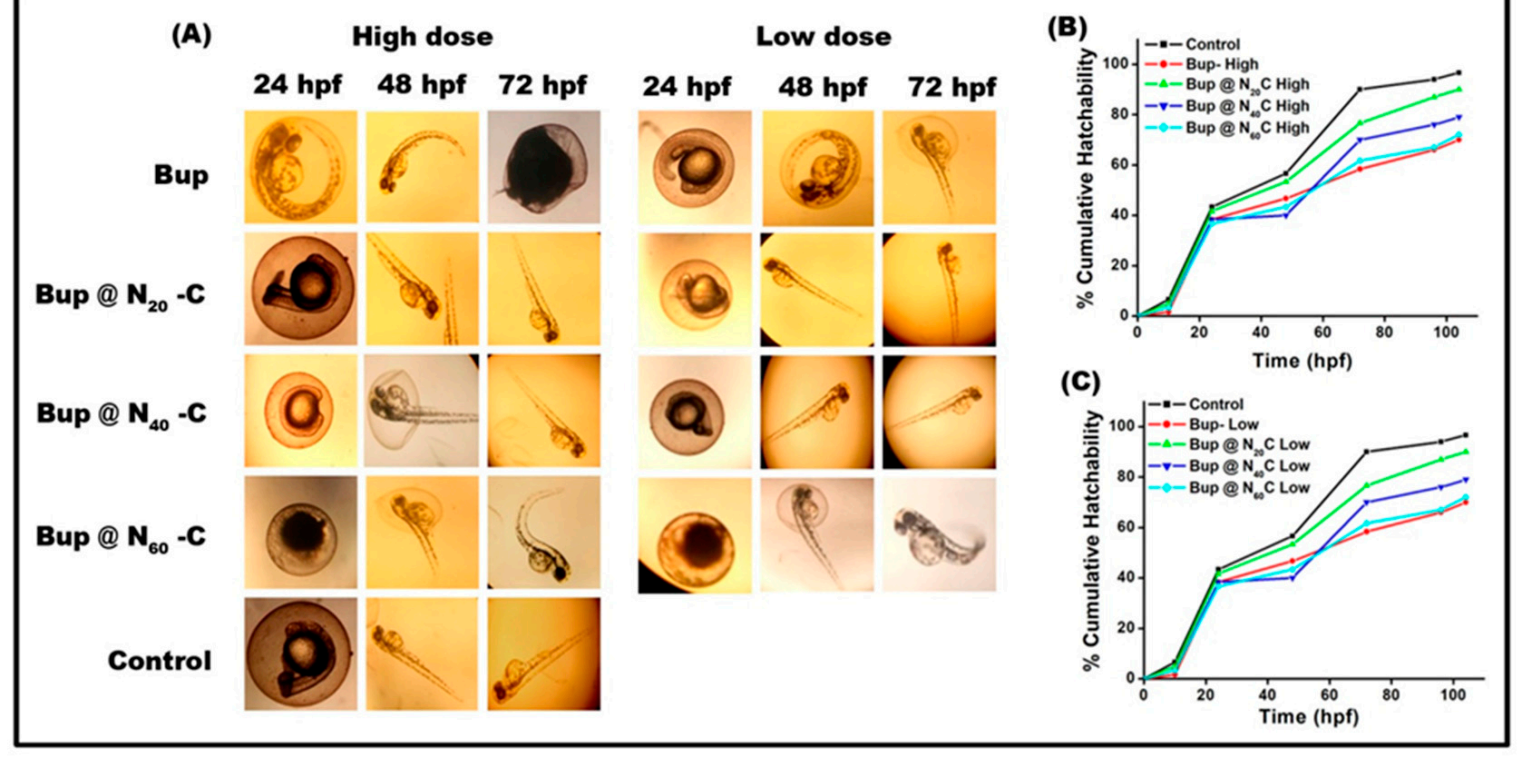
Developmental toxicity: (**A**) Observation of embryonic development of zebrafish under the microscope in the time intervals of 24, 48, and 59 hpf. The cumulative hatchability after treatment with high and low doses is represented in (**B**,**C**), respectively.

The toxicity of the formulation was evaluated using ZB embryos. Until 72 hpf, there was no developmental toxicity observed in the batch treated with a low dose of sample except Bup@N_60_C (Figure 12). A tail bend was observed in Bup@N_60_C at 72 hpf, which may be due to the presence of more drug molecules since the N_60_ vesicles had a high encapsulation efficiency. The hatchability of Bup@N_20_C was similar to that of the control, while the other groups showed decreased hatchability. Both in high and low doses of Bup@N_20_C, there was no notable developmental toxicity. However, the Bup@N_40_C and Bup@N_60_C formulations had delayed hatchability with tail bends. The percentage of hatchability of Bup@N_20_C was found to be 92% in high doses and 90% in low doses, while the control exhibited 96% of hatchability. So, the nanoformulated bupropion showed a minimal impact on hatching with absolutely no developmental toxicity.

## 3. Materials and Methods

Bupropion, reserpine, and surfactants (Span 20, Span 40, and Span 60) were purchased from TCI, Japan. Potassium chloride (KCl), sodium chloride (NaCl), calcium chloride (CaCl_2_·2H_2_O), disodium hydrogen phosphate (Na_2_HPO_4_·7H_2_O), and sodium bicarbonate (NaHCO_3_) were obtained from Rankem, while cholesterol, sodium dihydrogen phosphate (NaH_2_PO_4_·H_2_O), magnesium chloride (MgCl_2_·6H_2_O), ethylenediaminetetraacetic acid (EDTA), methanol, and dialysis membrane were procured from Himedia, Thane, India. Chloroform alone was obtained from SRL, India. For cell culture, Dulbecco’s Modified Eagle’s Medium (DMEM) and fetal bovine serum (FBS) were obtained from Gibco. The acridine orange (AO), ethidium bromide (EBr), and antibiotic solution were purchased from Hi-Media, Thane, India. All the chemicals are of analytical grade and used without any purification process. For all the experiments, we utilized double distilled water (d/w).

### 3.1. Preparation of Niosomes

Niosome with various surfactants was prepared in two batches using a thin film hydration technique as described in the previous study with slight modifications [42]. Mixture 1 containing surfactants (Span 20, Span 40, and Span 60) and cholesterol at various molar ratios was prepared as represented in Table S1 (Table S1 in Supplementary Materials). Mixture 2, consisting of chloroform and methanol, was taken in the molar ratio of 3:1. Both mixtures were transferred to a round-bottomed flask and subjected to a vacuum rotary evaporator to obtain a thin film. After drying, the flasks were placed in a desiccator containing silica gel overnight to ensure complete evaporation. The thin film was then hydrated with 10 mL d/w by soaking it for 2 h at room temperature and sonicating it for 15 min in the bath sonicator. The effect of varying concentrations of both cholesterol and surfactant on the optical density (OD), hydrodynamic diameter (D_h_), polydispersity index (PDI), and stability were studied. The obtained niosomal suspension was optimized and used for further analysis. The preparation of niosomes was performed in triplicates to avoid errors. 

### 3.2. Encapsulation of Antidepressant

Encapsulation of Bup in the niosomal vesicles was carried out using a sonication process to obtain monodispersive nanoformulations [58], for which 3 mL of the niosomal aqueous suspension and 5 µL of bupropion at the concentration of 10 mM were mixed in a beaker and sonicated using a bath sonicator for about 15 min at 25–30 °C at a frequency of 40 KHz for entrapment. 

### 3.3. Artificial CSF Recipe

The artificial cerebrospinal fluid (ACSF) was prepared following the previous study with slight modifications to suit the purpose [59]. The preparation involves mixing of solution A and solution B in the ratio of 1:1 where solution A consists of NaCl in a concentration of 296.33 mM, KCl of 6 mM, CaCl_2_·2H_2_O of 3.19 mM, and MgCl_2_·6H_2_O of 1.603 mM, while solution B consists of Na_2_HPO_4_·7H_2_O of 1.59 mM and NaH_2_PO_4_·H_2_O of 391.3 mM. For the mixture, autoclaved distilled water (d/w) was used. The sodium bicarbonate was excluded from the preparation protocol since it tends to shift the pH by converting it to carbon dioxide. This conversion can cause bubbles in the medium, which needs further processing steps.

### 3.4. Sample Characterization

For recording the UV-absorption spectra, the Shimadzu UV-1800 spectrophotometer was utilized. The hydrodynamic diameter (D_h_), polydispersity index (PDI), and zeta potential of the synthesized sample were analyzed using a Malvern Nano ZS-90 size analyzer. The diameter being measured pertains to the diffusion of a particle within a fluid, hence it is termed as a hydrodynamic diameter. This value represents the diameter of a sphere with an equivalent translational diffusion coefficient to the particle being analyzed. The translational diffusion coefficient of the particle is influenced not just by its core size but also by surface structures impacting diffusion speed, alongside factors like ion concentration and type in the surrounding medium. The zeta potential is measured by determining the electrophoretic mobility. The sample without any dilution was used for characterizing their D_h_, PDI, and their surface charge. The stability of the formulations was evaluated by recording the D_h_ and PDI every 7 days for a period of 28 days. The drug–vesicle interaction was studied using the Bruker-Alpha Fourier transform infrared (FTIR) spectrometer. The scanning electron microscope was employed to elucidate the shape, size, and morphology of the drug-entrapped vesicle. 

### 3.5. Encapsulation Efficiency (EE%)

The drug-encapsulated niosome was centrifuged for 15 min at 10,000 rpm using REMI RM-03 Plus Micro Centrifuge to obtain a clear supernatant. The supernatant was carefully removed and the concentration of the drug was determined using a UV spectrophotometer by obtaining optical density (OD) at 251 nm. The percentage of bupropion entrapped in the vesicles was calculated using the below formula [60].

$$EE\%=\frac{OD(i)-OD(s)}{OD(i)}\times 100$$

where *OD*(*i*) = initial OD of drug in the system and *OD*(*s*) = OD of supernatant.

### 3.6. In Vitro Drug Release Kinetics

The release pattern of bupropion from the niosomal vesicles in two different media was studied using the membrane diffusion method [61]. A freshly prepared 1× phosphate-buffered saline (PBS) pH = 7.4 and ACSF was chosen as the dissolution medium. After activation, the dialysis membrane was filled with 3 mL of the formulations and placed in a beaker containing 25 mL of PBS with the ends tied. The dissolution solution beaker was kept under continuous stirring throughout the experiment. A 2 mL solution from the beaker was withdrawn at time intervals of 15 min for 8 h and the OD was recorded at 251 nm. Soon after the withdrawal, a fresh 2 mL of PBS was added back to the beaker to maintain the final volume. The same protocol was repeated by replacing the PBS with ACSF as a dissolution medium. The OD of the samples was plotted against different time intervals to study the release profile of bupropion from the niosomal vesicles. 

### 3.7. Vesicle Stability Studies

The prepared vesicle was subjected to a study of the effect of temperature on its stability. The samples were taken in two glass containers and stored in two different temperature conditions—room temperature (25 °C) and refrigerator condition (4 °C). The D_h_ and PDI of the samples were measured every 7 days for a period of 28 days. 

### 3.8. Zebrafish Husbandry

Wild-type adult male and female zebrafish (ZB) (Danio rerio) were maintained in separate tanks provided with tap water in 12 h light–dark conditions. The temperature was maintained at 27 °C and the fish were fed with flakes twice a day. Once in two weeks, the fish were supplemented with live bloodworms. For embryo collection, male and female fish in the ratio of 2:1 were transferred to the breeding tanking and left overnight. The settled embryos were collected in a polystyrene petri plate, followed by washing with d/w using a pasture pipette to remove any debris. The healthy embryos were then used for the toxicity assessment [62]. All the experiments were conducted after acquiring clearance from the Institutional Animal Ethical Committee (IAEC). 

### 3.9. In Vitro Cell Viability Assay

The cytotoxicity of the niosomal vesicles before and after the drug encapsulation was determined using MTT assay on V79 and PC12 cells using a previously established protocol [63]. The cells were maintained in the DMEM with 1% antibiotic solution and 10% FBS at 37 °C in an incubator provided with 5% CO_2_ and a humidified atmosphere. Totals of 5.8 × 10^5^ and 7.6 × 10^5^ of V79 and PC12 cells per mL were seeded in a 48-well plate and subjected to incubation for 24 h. Upon incubation, the cells were treated with the prepared formulations in the concentration of 2, 5, 10, 15, and 20 µM, and further incubated for 24 h. The MTT at the concentration of 5 mg/mL was added in a dark condition to all the wells and incubated for 4 h. The formed formazan crystal was then dissolved using DMSO and the OD was recorded at 570 nm. The percentage of cell viability was calculated according to the procedure by Girigoswami et al. [64]. For assessing the viability of the cells, the dual staining method that involves AO and EBr at a concentration of 100 µg/mL was used. The photograph of the live and dead cells was captured using a fluorescence microscope. 

### 3.10. In Vivo Toxicity Assessment in Zebrafish Embryo

The protocol of the toxicity assessment was framed according to the previous study with slight modifications [65]. The ZB embryos were exposed to different concentrations (2, 10, and 20 µM) of bare and nanoformulated antidepressants. Thirty-five eggs were transferred to 6-well plates with 5 eggs per well. A total of three groups were made: a negative control group supplied with only E3 medium, a positive control group supplied with bupropion, and a study group. The study group further consisted of 3 batches, where one of the best formulations from each surfactant was chosen for the study. Drugs with different concentrations were tested over embryos to analyze the development-associated disorders in the first week of development. About 6 mL of E3 medium, with samples in each well-containing egg, was observed. The control eggs were also kept in the same condition. Every day, the medium was changed in all the wells. At 10 hpf, 24 hpf, 48 hpf, 72 hpf, 96 hpf, and 124 hpf, eggs were observed under an inverted microscope, and images were captured with the camera. The percentage cumulative hatchability was calculated for control and all the treated doses using the formula:

Cumulative hatchability %= (Eh/Ei) × 100

where Eh is the number of eggs hatched and Ei is the initial number of eggs taken. 

### 3.11. Hemolysis Assay

A hemolysis assay was performed to evaluate the hemocompatibility of the prepared samples following the protocol of the previous study with slight modifications [64]. The experiments were conducted following the ethical clearance from the Institutional Human Ethical Committee (IHEC)—registration no, IHEC-II/0432/23. An amount of 3 mL of blood was collected from a healthy volunteer and stabilized with ethylenediamine tetraacetic acid (EDTA). To obtain a pellet, the blood was centrifuged at 1500 rpm for 15 min. For the complete removal of serum particles, the pellet was washed with 1× PBS pH 7.4 and then diluted ten times with the same buffer. A 6 set of samples were prepared, where set 1 positive control contained 100 µL of the erythrocyte suspension with 900 µL of d/w, set 2 negative control contained 100 µL suspension with 900 µL of saline, and study samples contained 100 µL suspension with 900 µl of nanoformulated bupropion with Span 20 (set 3), Span 40 (set 4), and Span 60 (set 5). All the samples were incubated at 37 °C for 2 h and then centrifuged at 12,000 rpm for 1 min. The OD of the supernatant was measured at 251 nm and applied in the below formula for calculating the percentage of hemolysis.

% Hemolysis = (OD of study of sample − OD of negative control)/(OD of positive control − OD of negative control) × 100

### 3.12. Behavioral Analysis: Apparatus and Parameters

The behavioral analysis was conducted on five different tank setups—a novel test tank (NTT), color preference cross maze (CPM), dark/light (DL) box, open field test tank (OFT), and a social preference test tank (SPT)—by adapting the protocol from previous study with additional modifications [66,67]. A detailed description of the tank dimensions is illustrated in Figure S1 (Figure S1 in Supplementary Materials). Briefly, the NTT was sectioned into equal upper and lower halves from the level of water by marking outside the tank with a marker. The time spent by the ZB in each portion was recorded and analyzed. The CPM was made with four equal arms, covered with four different colors (blue, red, yellow, and green), while the junction point was kept transparent to introduce the fish into the tank. The DL box was sectioned into equal vertical parts to have dark and bright sections. The bright section was made by fixing a light source of CoB LED (chip-on-board light emitting diode) white light of 800 lumens in CoB high mode and the dark section was made by covering the tank with black origami chart paper. The OFT was a square tank with four equal sides without any partitions. The SPT was made by vertically sectioning the tank into 1:2:1 sections. The left section, called the conspecific region, was provided with 5 fellow fish, the middle section was used to introduce the fish, and the right section was kept empty. All the tanks were made up of transparent plexiglass. We made a simple video shooting setup with a tripod stand and a high-clarity mobile camera (OnePlus 9R) (Figure S2 in Supplementary Materials). The tanks were placed on a platform provided with a white background using origami chart paper. The video recording was conducted in a separate soundproof area to eliminate noise and other environmental interference. All the videos were recorded for 6 min and the analysis of trajectory data was performed using the software program ANY-maze. 

### 3.13. Behavioral Analysis: Methodology

The behavioral study was carried out to investigate the antidepressant activity of the prepared nanoformulation after obtaining ethical clearance from the Institutional Animal Ethics Committee (IAEC) (Approval No. 126/A.Lr:97). The experiment consisted of 6 sets of fish, including two sets of negative controls (Healthy fishes (Cn-a) and reserpine-induced depression (Cn-b)), one set of positive controls (stressed and treated with bupropion-Cp), and a study group consisting of 3 sets of stressed fishes treated with niosomes of three different surfactants nanoformulated with bupropion (NF1, NF2, and NF3). One best vesicle from the various concentrations per surfactant was chosen for the behavioral analysis. The fish was exposed to the tank containing 2 liters of reserpine at the concentration of 40 µg/mL for 20 min to induce acute depression. After administration, the fish were kept in five different tanks. The detailed study plan is illustrated in Figure S2. The video for Cn-b was recorded for 6 min after 7 days of 20 min of reserpine exposure. The Cp depression induction was treated with bare bupropion via oral administration using 10 µL pipette tips at a concentration of 20 µM. After the treatment, the fish were kept in a recovery tank for 5 min and then transferred to the previous storage tank. The video was recorded after 7 days since the onset of action of bupropion is around 1–2 weeks. The oral administration was performed after anesthetizing the fish using ice-cold water (8–10 °C). This same procedure is followed for the treatment and recording of all the other formulations. The changes in their locomotion, like preferences for color, light condition, distance traveled, speed, and time spent in the upper and lower part of the tank, were analyzed. All the experiment was conducted in triplicates.

### 3.14. Euthanasia of Zebrafish

The work is planned for further studies. Hence, after the treatment, the ZB were euthanized by adopting a rapid cooling method by submersing the larvae and fish in the container containing ice-cold water (0–4 °C) for 10 min and 25 min, respectively, to ensure death by hypoxia. Soon after euthanasia, the brains of the fishes were isolated and stored in 150 µL of 1× PBS pH 7.4 at −80 °C, after homogenization, using a micropestle for future analysis of neurotransmitter level. The carcasses of the fish and all the larvae were disposed of by incineration procedure.

### 3.15. Statistical Analysis

The statistical analysis was carried out using Prism Graphpad. The data are expressed as the mean ± SEM and were analyzed by one-way ANOVA followed by the Tukey post hoc test to find significant differences between the groups. Significance was defined as * *p* < 0.05, ** *p* < 0.01, *** *p* < 0.001, and **** *p* < 0.0001. 

## 4. Conclusions

Niosomes are being demonstrated as a favorable delivery system that could attain brain delivery of the cargo by escaping the BBB due to their tiny architecture. We have elucidated the potential of niosomes made of sorbitan ester surfactants via thin film hydration for encapsulating bupropion and delivering it to brain structures. After optimization, it has been found that the niosomes prepared with a cholesterol:surfactant concentration of 1:1.5 is the most stable system. We have also concluded that, among the three surfactants, niosomes with Span 20 (N_20_C) are the most stable formulation with minimal in vitro and in vivo toxicity, and exhibited sustained bupropion release in the ACSF medium. Through the behavioral analysis in the adult zebrafish model, we have elucidated that the bupropion carried in N_20_C vesicles is promising in improving mood by suppressing depression-like behavior. The Bup@N_20_C formulation increased the exploration activity and decreased irregular movements in the reserpine-induced depression model. The statistical analysis and the trajectory data revealed that the group treated with Bup@N_20_C (NF1) improved their mood more than the positive control group (bupropion treated-Cp) and similar to the control group (Cn-a). Being the first report on the nanoformulation of bupropion, we achieved a reversal of reserpine-induced depression behavior. However, this study only provided a preliminary conclusion and more significant clinical analyses that include the investigation of changes in the level of neurotransmitters are greatly required.

## Data Availability

Data is contained within the article and Supplementary Materials.

## Supplementary Materials

The following supporting information can be downloaded at: https://www.mdpi.com/article/10.3390/ph17030366/s1. Table S1: Niosome composition code to study the effect of varying concentrations of cholesterol and surfactant; Table S2: Average hydrodynamic diameter (D_h_), polydispersity index, and zeta potential of all the formulations; Table S3: Statistical data of the zebrafish locomotory patterns. The reserpine-induced behavioral alternations were significantly improved upon treatment with nanoformulated bupropion. Data are presented as mean ± SD; Figure S1: Representation of detailed description of the tank dimensions. The NTT was constructed with a width of 7 cm, the CPT had a 7cm height, the OFT had 20 cm of depth, the SPT had an 18 cm width, and the DL box had an 18 cm width; Figure S2: Experimental setup and methodology; Figure S3: Spectral representation of the hydrodynamic diameter of cholesterol optimized span 20 empty and bupropion-loaded vesicle.
