# Supplementary material for: Niosomal Bupropion: Exploring Therapeutic Frontiers through Behavioral Profiling
Source: Pharmaceuticals (Basel). 2024 Mar 12;17(3):366. doi: 10.3390/ph17030366 (PMC10976274; doi:10.3390/ph17030366)
Supplement: Supplementary file 1 [file pharmaceuticals-17-00366-s001.zip › pharmaceuticals-2905433-supplementary.pdf]

Article

Niosomal Bupropion: Exploring Therapeutic Frontiers through Behavioral Profiling

Karthick Harini[1], Suliman Yousef Alomar[2], Mohammed Vajagathali[1], Salim Manoharadas[3], Anbazhagan Thirumalai[1], Koyeli Girigoswami[1], Agnishwar Girigoswami[1*]

Table S1: Niosome composition code to study the effect of varying concentrations of cholesterol and surfactant.

Batch	Formulation code	Concentration of surfactant (mM)	Concentration of cholesterol (mM)	Methanol: chloroform	Concentration of drug (mM)
**Batch-1** Varying concentrations of cholesterol	*N_{20}A	1	0. 25	3:1	10
	*N_{20}B	1	0.5	3:1	10
	*N_{20}C	1	0.75	3:1	10
	*N_{20}D	1	1	3:1	10
	*N_{20}E	1	1.25	3:1	10
	*N_{20}F	1	1.5	3:1	10
	*N_{20}G	1	1.75	3:1	10
	*N_{20}H	1	2	3:1	10
**Batch-2** Varying concentrations of surfactant	N_{20}A, N_{40}A, N_{60}A	0. 5	1	3:1	10
	N_{20}B, N_{40}B, N_{60}B	1	1	3:1	10
	N_{20}C, N_{40}C, N_{60}C	1.5	1	3:1	10
	N_{20}D, N_{40}D, N_{60}D	2	1	3:1	10
	N_{20}E, N_{40}E, N_{60}E	3	1	3:1	10

N_{20} represents the series of vesicles containing span 20 surfactants, N_{40} represents the series of vesicles containing span 40 surfactants, and N_{60} represents the series of vesicles containing span 60 surfactants.

Table S2: Average hydrodynamic diameter (D_h), polydispersity index, and zeta potential of all the formulations

Formulation code	Hydrodynamic diameter (nm)	Zeta potential (mV)	Polydispersity index (PDI)
N_{20}A	189.0 ± 4	-32.4 ± 2	0.502 ± 0.04
N_{20}B	255.7 ± 3	-33.2 ± 6	0.491 ± 0.01
N_{20}C	365.1 ± 3.5	-38.1 ± 1	0.448 ± 0.02
Bup @ N_{20}C	344 ± 4.2	-38.4 ± 4	0.480 ± 0.02
N_{20}D	286.9 ± 2.6	-23.5 ± 2	0.65 ± 0.01
N_{20}E	400.2 ± 4.8	-30.9 ± 2	0.550 ± 0.02
N_{40}A	183.7 ± 4	-29.8 ± 3	0.447 ± 0.04
N_{40}B	229.1 ± 4	-34.0 ± 2	0.343 ± 0.01
N_{40}C	256 ± 3.8	-27.9 ± 4	0.349 ± 0.10
Bup @ N_{40}C	249 ± 6	-34.7 ± 2	0.295 ± 0.09
N_{40}D	198.9 ± 7	-32.4 ± 2	0.368 ± 0.08
N_{40}E	299.5 ± 4	-29.0 ± 5	0.264 ± 0.04
N_{60}A	187.4 ± 8	-28.8 ± 2	0.248 ± 0.02
N_{60}B	182.8 ± 6	-28.5 ± 2	0.304 ± 0.01
N_{60}C	243.2 ± 3	-36.6 ± 2	0.425 ± 0.04

Bup @ $N_{60}C$	276.6 ± 3.9	-36.3 ± 3	0.258 ± 0.03
$N_{60}D$	169.2 ± 6	-25.3 ± 4	0.275 ± 0.06
$N_{60}E$	273.7 ± 5.4	-34.8 ± 2	0.471 ± 0.02

Table S3: Statistical data of the zebrafish locomotory patterns. The reserpine-induced behavioral alternations were significantly improved upon treatment with nanoformulated bupropion. Data are presented as mean ± SD.

Behavioural tests	Parameters	Groups						F (DFn, DFd)	*P* value
		Cn-a	Cn-b	Cp	NF1	NF2	NF3		
**Novel tank test**	Upper Zone (s)	193 ± 7.48	74 ± 8.04	154.3 ± 6.94	174 ± 6. 68	113 ± 6.53	129.3 ± 4.49	F (5, 12) = 122.0	*P*<0.0001
	Lower Zone (s)	167 ± 7.48	286 ± 8.04	205.6 ± 6.94	186 ± 6.68	247 ± 6.53	230.6 ± 4.49	F (5, 12) = 122.0	*P*<0.0001
**Open field test**	Distance travelled (m)	125 ± 5.35	67.3 ± 9.80	104.6 ± 6.94	136.3 ± 6.12	86.3 ± 7.84	96 ± 6.68	F (5, 12) = 36.31	*P*<0.0001
	Average speed (m/s)	0.387 ± 0.008	0.177 ± 0.009	0.292 ± 0.004	0.388 ± 0.005	0.304 ± 0.005	0.293 ± 0.004	F (5, 12) = 405.2	*P*<0.0001
**Social preference test**	Conspecific region (s)	305.6 ± 7.03	46.6 ± 6.54	49.6 ± 4.10	250.6 ± 4.49	150.6 ± 4.49	169 ± 6.53	F (5, 12) = 1021	*P*<0.0001
	Middle region (s)	22.6 ± 3.29	80.3 ± 7.36	282.3 ± 4.98	61.3 ± 4.10	85.6 ± 5.43	79.3 ± 7.36	F (5, 12) = 787.7	*P*<0.0001
	Empty region (s)	31.6 ± 6.18	233 ± 6.16	28 ± 7.11	48 ± 7.78	123.6 ± 7.13	111.6 ± 7.36	F (5, 12) = 379.4	*P*<0.0001
**Colour preference test**	Blue zone (s)	259.3 ± 7.40	48.6 ± 5.31	72 ± 6.97	263 ± 6.16	174.6 ± 6.94	77.3 ± 7.93	F (5, 12) = 742.9	*P*<0.0001
	Green zone (s)	51.3 ± 4.49	29.6 ± 7.36	33.6 ± 5.24	15.6 ± 3.86	51.6 ± 7.71	127.3 ± 5.79	F (5, 12) = 15.1	*P*<0.0001

	Yellow zone (s)	28 ± 4.49	95.3 ± 6.64	134.3 ± 6.94	9 ± 1.63	24.6 ±5.31	22 ± 6.16	$F_{(5, 12)} = 251.8$	$P<0.0001$
	Red zone (s)	21.3 ± 7.09	186.3 ± 15.10	120 ± 8.83	72.3 ± 5.90	109 ± 4.32	133.3 ± 6.18	$F_{(5, 12)} = 126.3$	$P<0.0001$
**Dark/light box**	Dark region (s)	228 ± 5.71	115 ± 4.89	176.3 ± 4.10	231.6 ± 4.92	188.6 ± 6.12	164 ± 6.16	$F_{(5, 12)} = 196.3$	$P<0.0001$
	Light Zone (s)	132 ± 5.71	245 ± 4.89	183.6 ± 4.10	128.3 ± 4.92	171.3 ± 6.12	196 ± 6.16	$F_{(5, 12)} = 196.3$	$P<0.0001$

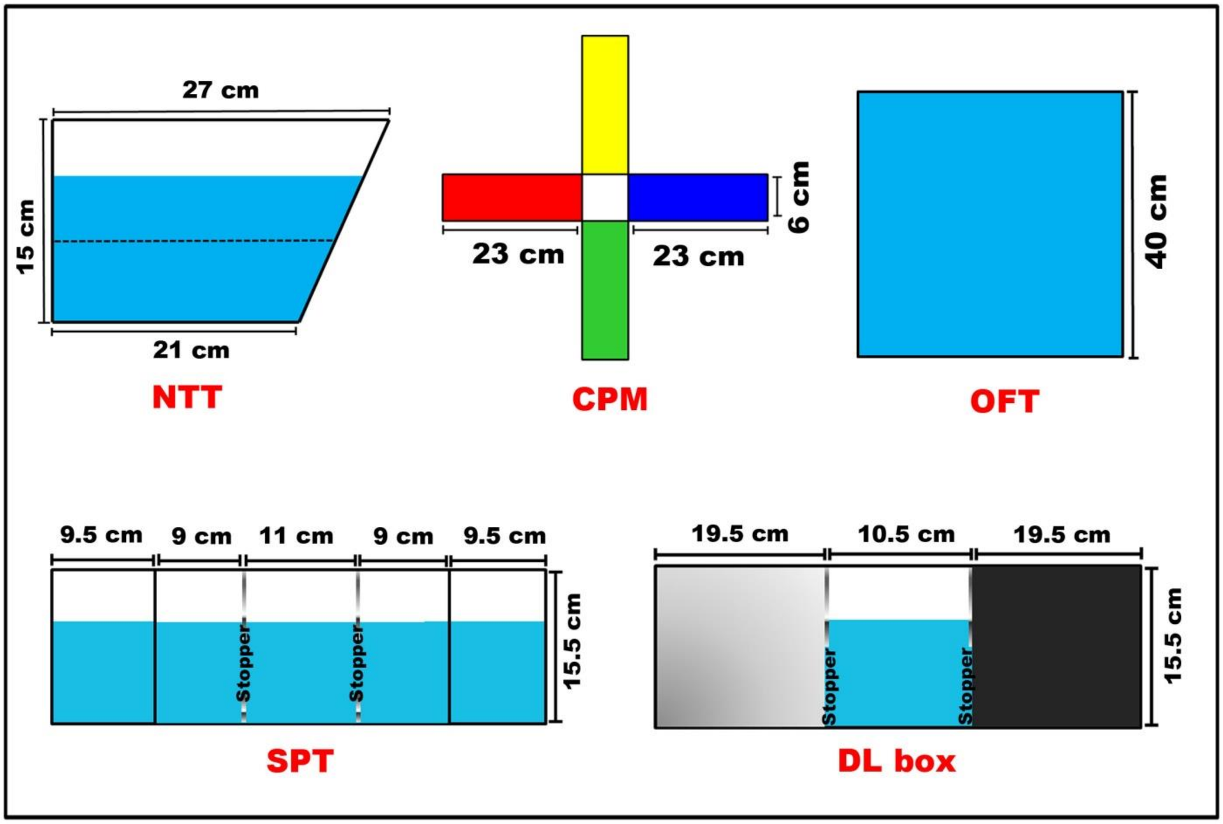

Figure S1: Representation of detailed description of the tank dimensions. The NTT was constructed with a width of 7 cm, the CPT had a 7cm height, the OFT had 20 cm of depth, the SPT had an 18 cm width, and the DL box had an 18 cm width.

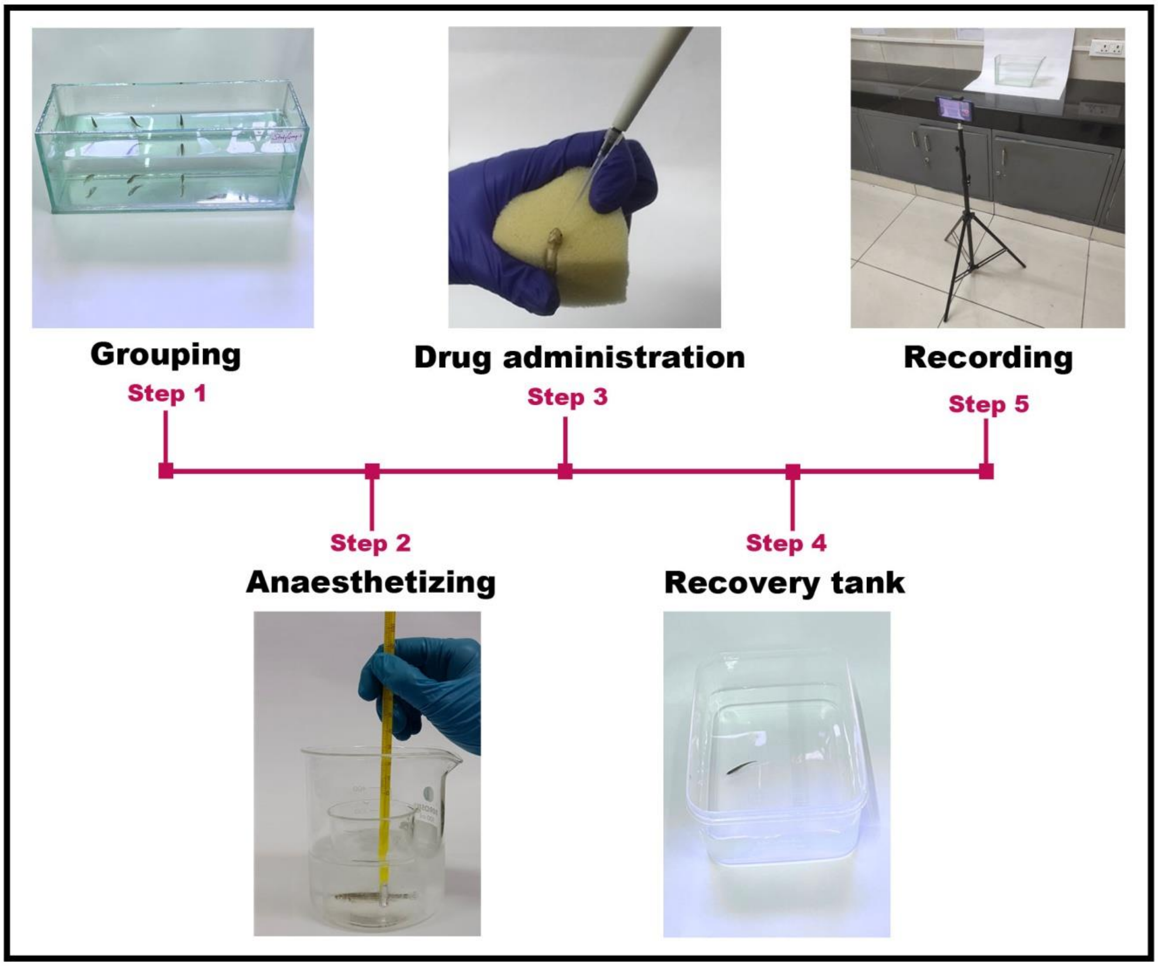

Figure S2: Experimental setup and methodology

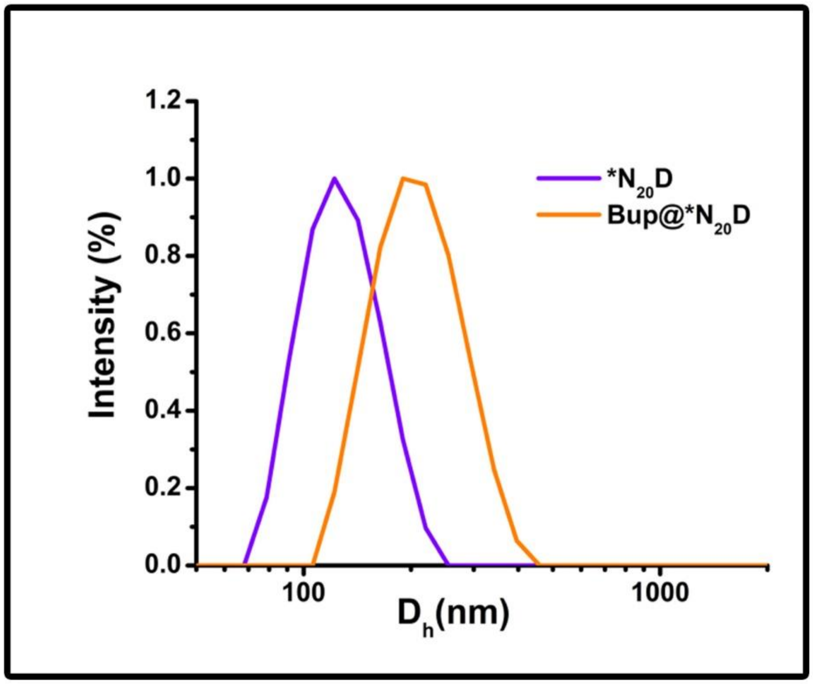

Figure S3: Spectral representation of the hydrodynamic diameter of cholesterol optimized span 20 empty and bupropion-loaded vesicle
